# Stream-Based Visually Lossless Data Compression Applying Variable Bit-Length ADPCM Encoding

**DOI:** 10.3390/s21134602

**Published:** 2021-07-05

**Authors:** Shinichi Yamagiwa, Yuma Ichinomiya

**Affiliations:** 1 Faculty of Engineering, Information and Systems, University of Tsukuba, 1-1-1 Tennodai, Tsukuba, Ibaraki 305-8573, Japan; 2 JST, PRESTO, 4-1-8 Honcho, Kawaguchi, Saitama 332-0012, Japan; 3 Department of Computer Science, University of Tsukuba, 1-1-1 Tennodai, Tsukuba, Ibaraki 305-8573, Japan; ichinomiya@padc.cs.tsukuba.ac.jp

**Keywords:** data compression, ADPCM, visual data compression, stream-based data compression, lossless data compression, ASE coding

## Abstract

Video applications have become one of the major services in the engineering field, which are implemented by server–client systems connected via the Internet, broadcasting services for mobile devices such as smartphones and surveillance cameras for security. Recently, the majority of video encoding mechanisms to reduce the data rate are mainly lossy compression methods such as the MPEG format. However, when we consider special needs for high-speed communication such as display applications and object detection ones with high accuracy from the video stream, we need to address the encoding mechanism without any loss of pixel information, called visually lossless compression. This paper focuses on the Adaptive Differential Pulse Code Modulation (ADPCM) that encodes a data stream into a constant bit length per data element. However, the conventional ADPCM does not have any mechanism to control dynamically the encoding bit length. We propose a novel ADPCM that provides a mechanism with a variable bit-length control, called ADPCM-VBL, for the encoding/decoding mechanism. Furthermore, since we expect that the encoded data from ADPCM maintains low entropy, we expect to reduce the amount of data by applying a lossless data compression. Applying ADPCM-VBL and a lossless data compression, this paper proposes a video transfer system that controls throughput autonomously in the communication data path. Through evaluations focusing on the aspects of the encoding performance and the image quality, we confirm that the proposed mechanisms effectively work on the applications that needs visually lossless compression by encoding video stream in low latency.

## 1. Introduction

The video data stream is one of main data streams utilized in the recent mobile and Internet of Things (IoT) applications. For example, surveillance camera systems [1] are widely used to detect irregular events in society [2,3] and home security [4] by applying machine learning methods [5,6]. In such applications, a fast, seamless and high resolution video transmission improves the accuracy of the image processing for the video frames. We can also find hardware sensory approaches for accelerating image processing such as the dynamic vision sensor [7] applied to applications (see, e.g., [8]) and the retina-like sensor [9]. However, the main technology trend of the video image processing is still based on the techniques for high performance processing of frame data stream captured from commodity color image sensors.

Recent methods to transfer the video data stream focus on maintaining an available data rate between the producer and the consumer. The main objective is to keep a stable casting of the video stream in the communication media. For example, the Moving Picture Experts Group (MPEG) format uses a discrete cosine transform to reduce high- and low-frequency parts in the luminance and color of frames. Inserting key frames periodically, the technique compliments differences among the frames. This kind of video transfer method is used in broadcasting on digital TVs and video streaming services on the Internet such as via web cameras and smartphones. Because the information of original image frames is reduced, we call the method to compress video data stream *lossy compression*.

In the lossy compression system, effective architectures are proposed (e.g., [10]), which handles multiple connections from the producer to the consumers and the ones with unstable bandwidth. The architecture uses the buffering technique to maintain stable data communication. The buffering is effective to keep a stable transfer of video data stream not to drop frames. However, it increases delay from the production of the original image data to the visualization on the consumer side.

Although the lossy compression method is effective to provide a stable bandwidth of video data stream, it needs large calculations for deriving statistics of video stream that can be removed from the original color pixels. The recent human-less automation lines and IoT applications in industry demand fast processing for high-yield production. The major examples are image inspection [11] and security equipment using video images [1]. Those need to reduce latency among the processes for image acquisition, the transfer of the image to the abnormal detection and the control to exclude the abnormal state [12]. The final process feedbacks the action from the detection processor to the mechanical control. The higher feedback speed will result in higher yield production. Here, we can accelerate the abnormal detection because we can apply high performance processors on the server side with Graphics Processing Units (GPUs) [13] and Artificial Intelligence (AI) processors [14]. On the other hand, the communication part of the system includes the lossy compression and the transfer via a network media. Even if we improve the communication performance of the network, the lossy compression part will become the bottleneck to transfer the video stream to the abnormal detection part due to the heavy calculation of the compression processes. To overcome the difficulty of improving the lossy compression performance, we can accelerate the compressor and the decompressor by applying GPUs [15] and dedicated hardware [16]. However, the lossy compression reduces information of pixels in frames and guarantees the bandwidth of the video stream. This inevitably lacks small objects in the frame and degrades the abnormal detection performance. Thus, we need to develop a novel lossy compression mechanism that maintains pixel level information in video frames and achieves a controllable bandwidth of the video stream with a small delay.

To overcome the low latency compression and manage the stable transmission of video data stream, a compression method was proposed equipped in High-Definition Multimedia Interface (HDMI) [17]. The resolution of display is enormously growing from HD to 4K and 8K. The large resolution and the high frame rate cause difficulties for image data transmission. The Video Electronics Standards Association (VESA) proposed a solution for the situation, called Display Stream Compression (DSC) [18]. This standard includes a data compression technique that applies Differential Pulse-Code Modulation (DPCM) to the video data stream. DPCM reduces the number of bits in a pixel by quantizing differences between color data of the neighbor pixels in a video frame. If the number of the quantized bits is less than the one of the original data, it can compress each pixel. Because the method uses simple calculations, we can implement it in a fast software/hardware without buffering. Thus, DSC is able to compress the video data stream and also can compress the original video data stream. However, DPCM can result in overranges when the difference among the pixels is large. To avoid this overrange situation, we need to employ a larger number of bits for the quantization.

The compression method based on DPCM replaces the original pixels with the quantized color values based on the differences among the neighbor pixels. The color information is visually acceptable even if the difference overranges the number of encoding bits used by the quantization. In this paper, we call this lossy compression method that reserves all quantized/original pixel data of video frames *visually lossless* data compression. It has an advantage that the compressed data maintains the full information of all pixels. However, considering the implementation of industrial applications, we need to address the aspects of the processing speed of treating the video stream and the accuracy of the predicted pixel color. Thus, according to the discussion above, we need to develop a novel method for visually lossless compression that satisfies the following three conditions: (a) it reserves visually equivalent pixel color information; (b) it processes video data stream without stalling and in low latency; and (c) it controls bandwidth adaptively against the dynamic overhead of the communication data path for sending the compressed video data.

This paper proposes a new method of visually lossless compression applying the Adaptive Differential Pulse-Code Modulation (ADPCM) [19]. ADPCM is a classical method extended from DPCM to calculate the compressed data adaptively from predicted difference value among the original data. ADPCM is mainly and widely used for compression of Pulse-Code Modulation (PCM) data in audio applications such as wireless microphones and headphones. This paper applies the method to pixel data of video frames and validates the effect of ADPCM in video application. Although ADPCM can predict adaptively pixel colors in a number of compressed data bits, the number is fixed by a configuration of parameters in ADPCM. If the number of compressed data bits *M* is smaller than the one of the original color data *N*, it constantly compresses to M/N times. This is not suitable for the dynamic bandwidth in communication data path. In this paper, we also propose a new ADPCM with variable bit-length mechanism. Additionally, we expect that the compressed data after visually lossless compression maintain low entropy due to the algorithm of ADPCM. We also apply lossless data compression after ADPCM with variable bit length. This provides further compression effect to the video stream. By applying the stream-based lossless data compression called Adaptive Stream-based Entropy (ASE) coding [20] with visually lossless compression, we propose a video transfer system that implements dynamic control of the data transfer in communication data path.

The main contributions of this paper that can be used for other related systems are as follows:

1. We developed a new ADPCM by adding a mechanism for variable bit length in the conventional method. It is able to control the compressed data size and the image quality dynamically. The video image quality is acceptable in the industrial applications.
2. We proved that a lossless data compression is effective by applying it after the proposed ADPCM with variable bit length. Applying our previous work, ASE coding, we evaluated the effect of the lossless data compression by experimental evaluation.
3. We proposed a low latency video transfer system by combining the novel ADPCM with variable bit length and a lossless data compression. We employed our previous work, ASE coding, to implement the stream-based manner. The system works without any stalling during the data compression and achieves low latency for video data stream. We proved the validity of the system from experimental evaluations by software emulation.

This paper is organized as follows. Section 2 describes the backgrounds and the definitions of our research. Section 3 proposes the method for ADPCM with variable bit length and will organize a video transfer system. Section 4 evaluates and validates the compression performances of the proposed visually lossless compression method and the system. Finally, we conclude the paper.

## 2. Background and Definitions

### 2.1. Visual Data Compression

The image compression techniques have been given attention by the field of signal processing [21]. Many techniques to overcome Shannon’s limit have been proposed and implemented such as the data compression technique that removes high and low frequencies in the image applying Fast Furrier Transform (FFT) and Discrete Cosine Transform (DCT). These lossy methods predict pixels in images from the reduced frequency information. JPEG2000 is one of the well-known methods applying wavelet transform [22]. It decodes the frequency information to pixel colors by a wavelet. The wavelet transform can also provide a lossless compression method. Although the compressed data can become larger than the data size of the original image, it is mainly utilized as a lossy compression method. We can decide decoding overhead by selecting the frequency levels encoded by the method of the wavelet transform. This allows us to choose a tradeoff between the processing performance and the image quality. These methods were originally developed for still image data to compress them considering portability among information equipment. However, the encoders for the methods need to scan whole image pixels with buffering the color information as well as a large amount of computation to compress multiple image frames in the timeline of a video. Therefore, novel image and video compression methods have been developed.

Due to the growth of streaming broadcasting via the Internet, it is standardized to perform streaming of movies by using the MPEG format. The format provides encoding methods with the frequency-based compression mentioned above and another method in the timeline direction. The key frame is introduced in the format, which is a compressed full frame image data. The format encodes multiple frames in the timeline direction by extracting the differences of pixel color information from the key frame. The method provides techniques to compliment the differences based on the visual nature that human eyes do not sense precisely the high-frequency parts of scenes. Therefore, the format reduces the color information between the key frames. The motion detection techniques (e.g., [23]) help to compliment the color and luminance information to chase changes of color pixels in video using vectors derived from the color changes. This technique results in a good compression ratio in the encoded movie data. Thus, the methods explained above can transfer the movie via an unstable communication data path by controlling throughput of the compressed data size.

Advanced video encoders such as H.264 and H.265 [24] have recently been utilized for embedded small devices in the fields of IoT and mobile devices [25]. However, we need to address two difficulties in the implementations of the embedded applications. The first one is the degradation of image quality because the number of predicted pixels increases as the data size of the encoded video stream is reduced. The performance of machine learning applications such as object detections can degrade due to the lower image quality. To maintain good performance for the detection, we need to decide to include enough key frames. Therefore, we need to consider the tradeoff between the video quality and the compression ratio [26]. Another difficulty is the overhead for encoding video data as increasing the sort of compression algorithms such as the motion detections. Those algorithms need to buffer and scan multiple frames of the original video. Although these dense compression processes provide good compression ratio, increasing the encoding time becomes a drawback for realtime processing [27,28]. Therefore, we need to invent a novel video encoding method that overcomes these two difficulties. It provides a fast and compact implementation particularly in embedded systems such as sensor networks [4], surveillance cameras [1,3] in security IoT equipment [2] and applications in factory automation [11,29].

### 2.2. Visual Lossless Compression Methods

**Figure 1:**
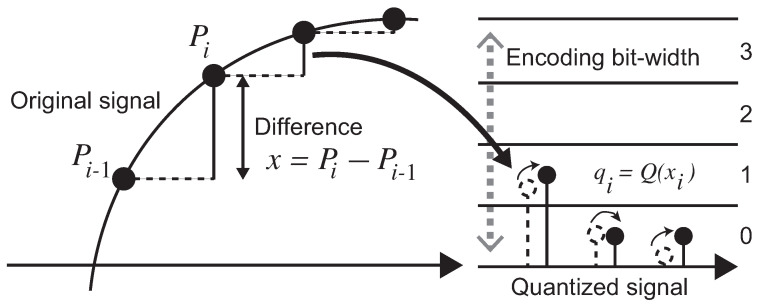
Encoding processes of DPCM.

DPCM was applied originally to the audio data compression field. The DSC compresses a video stream that consists of pixel color data as a single data stream by applying DPCM. As shown in Figure 1, DPCM processes a pixel data stream $P_{i}$ of a *Width* × *Height* image frame, which data width of each pixel is *N* bit. DPCM calculates the difference $x_{k}$ between $P_{i-1}$ and $P_{i}$ and quantizes it to an *M* bit value $q_{k}$. The encoding flow is formally written as follows;

$$x_{i}=P_{i}-P_{i-1}$$

$$q_{i}=Q\left(x_{i}\right).$$

Here, when the condition M<N is satisfied, the method reduces the amount of the original pixel data stream. The decoding is easily performed by the reverse calculations of the encoding steps: $q_{i}$ is dequantized by the offset used during the quantization and then the result is added to the previous pixel value $P_{i-1}$.

During the encoding and the decoding processes of DPCM, it only needs to buffer single pixel data. Therefore, it can be implemented in software/hardware with small resources and runs quickly because the processing steps are very simple. Therefore, it works effectively in a stable communication data path in the applications. In the aspect of image quality, DPCM maintains all pixels nearly equivalent to the originals if we can apply an effective quantization for visually equality. In this paper, we call this kind of method that reserves whole nearly equal pixels *visually lossless* compression. However, unless DPCM applies an *M* that consists of a larger number of bits not to cause overflow during the quantization, the pixel quality degrades due to its coarse resolution. Therefore, we need to decide the bit length of *M* heuristically with considering the tradeoff between the image quality and the compression ratio statically.

### 2.3. ADPCM

Adaptive DPCM (ADPCM) [19] is another method for visually lossless compression that overcomes the problem on the tradeoff regarding bit length of *M*. ADPCM was developed in the 1980s mainly for audio data compression targeted to telephone and fax. It is also standardized by ITU Telecommunication Standardization Sector [30]. ADPCM uses an adaptive quantization mechanism that follows data tendency in *M* bits.

**Figure 2:**
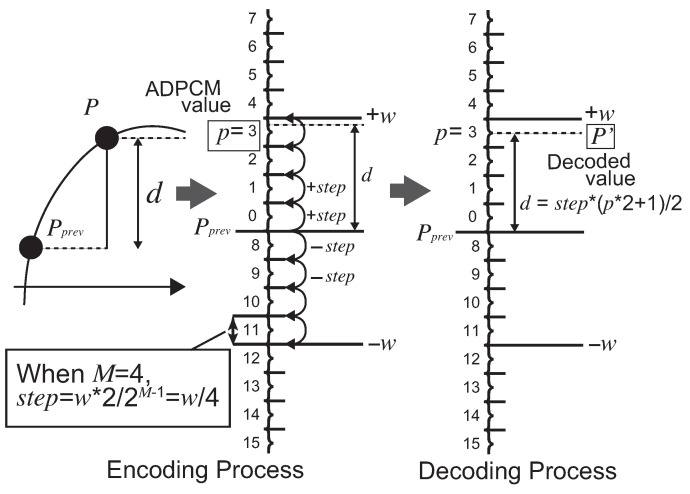
Encoding and decoding processes of ADPCM.

Let us explain the algorithm for a typical implementation of ADPCM by referring to Figure 2. Here, let us consider the case when an original unit of a data stream $P_{k}$ in *N* bits is compressed (encoded) to an ADPCM value $p_{i}$ in *M* bits. We show the encoding steps in Algorithm 1. The figure depicts the roles of parameters and values combining with an example where N=8 and M=4. That performs a quantization of $p_{i}=Q\left(P_{i}\right)$. The condition N>M satisfies to compress the original data stream. At compressing the *i*th original data $P_{i}$ to an ADPCM value $p_{i}$ in a data stream, the encoding can be invoked by caching the previous original data $P_{i-1}$ and the *predicted offset* $w_{i-1}$ that has been used for deriving $P_{i-1}$. The first step of the algorithm calculates the difference $d_{i}=P_{i}-P_{i-1}$. Then, the next step is the important part of the encoding to derive the ADPCM value. The encoder maps region numbers in *M* bits regarding $d_{i}$ to the predicted domain specified in an axis between $+w_{i-1}\times 2$ and $-w_{i-1}\times 2$. The domain is divided into $2^{M}$ regions. The region numbers are assigned as incremental numbers, as illustrated in Figure 2. Here, the regions more than $+w_{i-1}\times 2$ and less than $-w_{i-1}\times 2$ are assigned to $2^{M-1}-1$ and $2^{M}-1$, respectively. Finally, the region number of $d_{i}$ is outputted as the ADPCM value $p_{i}$. The $p_{i}$ is a positive integer value and satisfies the condition $0\leq p_{i}\leq 2^{M}-1$. The predicted offset is updated by a function $f(w_{i-1},p_{i})$ for the next encoding. The function is specified heuristically, for instance:

$$w_{i}=f\left(w_{i-1},p_{i}\right)=w_{i-1}\times C\left(p_{i}\right)/\left(2^{M}\times 4\right).$$

The *C* is a constant value of an array associated by the M−1 bits of $p_{i}$. The array is configured by, for example, {57, 57, 57, 57, 77, 102, 128, 153} where M=4. These values are actually used in some frequency modulation sound ICs. The function *f* returns the larger value when the difference *d* becomes larger. This means that the predicted domain is assigned in a wider area when the original data change largely. Therefore, the encoding works for the quantization by avoiding overrange and follows the value change of $P_{i}$ adaptively in the timeline. Regarding an implementation of Algorithm 1, $f_{prev}$ and *w* are equivalent. We only need to cache $P_{prev}$ and *w* during the encoding steps.
**Algorithm 1** Encoder of ADPCM [19].**Require:***P* // a unit of data to be compressed. **Ensure:**
*p* // an ADPCM value. 
w←
W_INIT 
$P_{prev}\leftarrow$
P_INIT 
$C\left[\right]\leftarrow \left[c_{0},c_{1},...,c_{2^{M-1}}\right]$ 
$f_{prev}\leftarrow w$ 
$d\leftarrow P-P_{prev}$ 
$step\leftarrow \left(w\times 2\right)/2^{M-1}$  **if**
d>0
**then**  **if**
$\left|d\right|/step>2^{M-1}-1$
**then**   
$p\leftarrow 2^{M-1}-1$  **else**   
p←ceil(d/step)  **end if**  **else**  **if**
$\left|d\right|/step>2^{M}-1$
**then**   
$p\leftarrow 2^{M}-1$  **else**   
$p\leftarrow ceil\left(-d/step\right)+2^{M-1}$  **end if** **end if** 
$f_{prev}\leftarrow f_{prev}\times C\left[p\&\left(\left(1\ll M\right)-1\right)\right]/64$ 
$w\leftarrow f_{prev}$


The decoding performs $P_{i}=Q^{-1}\left(p_{i}\right)$, as listed in Algorithm 2. The decoder requires the predicted original value $P_{i-1}$ and a predicted offset $w_{i-1}$ in the previous decoding. In addition to the encoding steps, the decoder outputs a predicted original value $P_{i}$ by using the middle value in a region selected from $p_{i}$. Similar to the encoding steps, the region is derived by using $w_{i-1}$ divided into $2^{M}$ regions, as depicted in Figure 2. Then, $w_{i}$ is updated by the equivalent function $f(w_{i-1},p_{i})$ used in the encoder. Here, the decoder also needs to cache just $P_{i-1}$ and $w_{i}$.

As explained above, ADPCM does not need to buffer a large amount of data such as an image frame or a line in a frame. Furthermore, it encodes and decodes with simple calculations. It also works quickly on a hardware implementation with little resources. According to the characteristics of the algorithm, we are able to implement a fast visually lossless compression method. Actually, advanced studies (e.g., [31,32,33]) are applying ADPCM to embedded systems and IoT applications as an image compression methods. Although we can find the other standardized methods (e.g., [34,35]), those need to buffer one or more lines during encoding and decoding steps. This is a potential drawback to accept a video data stream in an implementation with minimal resources as ADPCM performs.
**Algorithm 2** Decoder of ADPCM [19].**Require:***p* // an ADPCM value. **Ensure:**
*P* // a unit of data to be compressed. 
w←
W_INIT 
$P_{prev}\leftarrow$
P_INIT 
$C\left[\right]\leftarrow \left[c_{0},c_{1},...,c_{2^{M-1}}\right]$ 
$f_{prev}\leftarrow w$ 
$step\leftarrow \left(w\times 2\right)/2^{M-1}$  **if**
$p<2^{M-1}$
**then**   
d←step×(p×2+1)/2  **else**   
$d\leftarrow -step\times (\left(p-2^{M-1}\right)\times 2+1)/2$  **end if**  
$P\leftarrow P_{prev}+d$  
$f_{prev}\leftarrow f_{prev}\times C\left[p\&\left(\left(1\ll M\right)-1\right)\right]/64$  
$w\leftarrow f_{prev}$


In ADPCM, a larger *M* results in better image quality. The number of bits is fixed to a constant one conventionally. Therefore, we must decide the best *M* to satisfy the application’s needs heuristically. However, this does not allow us to apply ADPCM to any applications that transfer images via an unstable communication data path or on memory devices with dynamic throughput.

### 2.4. Stream-Based Lossless Data Compression

Another style of data compression method is the lossless method that the original data are decoded from the compressed ones. Focusing on the lossless compression for data stream, the Adaptive Stream-based Entropy (ASE) coding has been developed [20,36]. It eliminates buffering any part of the data stream and any stall during compression/ decompression operations based on a look-up table. Because we can choose any number of the table entries, it is available to implement the compressor/decompressor using few software/hardware resources.

ASE coding compresses an *N* bit original data unit in a data stream (called *symbol*) using the lookup-table. When the symbol is hit in an index of the table, the index is outputted as the compressed data with the Cmark bit (=1) by reducing it to *m* bits with the entropy calculation $m=\log_{2}k$, where *k* is the number of occupied entries in the table. On the other hand, when the symbol is missed in the table, it is registered to the table and the original symbol is outputted with the Cmark bit (=0). When the decompressor receives the compressed data stream from the compressor, it first receives the Cmark bit and checks if the subsequent data bits are compressed or original. If the Cmark is set, the subsequent *m* bits calculated by the same entropy calculation using the number of occupied entries in the decompressor. The *m* bis are extended to the number of bits of the table index with zero(s). Then, the decompressor associates the contents of the table by the index and outputs the symbol from the table. If the Cmark is zero, the subsequent *N* bits are registered to the look-up table and outputs it as the original symbol.

Here, the look-up tables in both the compressor and the decompressor are equivalently managed because the registrations of new symbols are performed at the same timings. The look-up table is managed as a stack in the Least Recently Used (LRU) manner. When the symbol is hit in the table, the associate entry is moved to the top of the table. Otherwise, the new symbol is pushed from the top. This mechanism allows the compressor/decompressor to know the number of occupied entries *k*.

As the operations proceed, the table will become full. In this case, *k* becomes always the number of table entries *E*. This means that the compressed data size equals *M* = $\log_{2}E$ and then the compression does not work. To avoid this situation, ASE coding has a mechanism to delete occupied entries in the look-up table from the one in the bottom, called the *entropy culling*. After a number of table hits during the compression/decompression, it removes an occupied entry at the bottom of the table. This mechanism adaptively follows data entropy of data stream and instantaneously assigns the minimal number of bits as the compressed data.

As shown above, ASE coding works with simple operations without buffering as accepting data stream. Thus, it is implemented on fast software/hardware.

Here, let us consider a situation with lossless data compression when the compressor conveys a change of configuration to the decompressor. This occurs when, for example, the bit width of a symbol changes in the compressor and it must be conveyed to the decompressor. The conventional algorithms such as LZ-based methods [37] prepare special symbol for the exception handling. However, this needs to allow the compressor to waste additional bits to the compressed data. Then, the compression ratio becomes worse. To avoid this situation, ASE coding introduces the *exception symbol* [38] that provides the implicit information in the compressed data stream without any additional bits. The mechanism works with inconsistent states that occur when the table search operation meets inconsistent result. The exception symbol consists of two types: (1) an original symbol with Cmark = 0 that is already registered in the look-up table; or (2) a compressed symbol with Cmark = 1 that is an index of an empty entry in the table. The former exception symbol is sent from the compressor, and the decompressor tries to register the symbol. However, an inconsistent state occurs because it is already registered in the table. Besides, in the case of the latter exception symbol, the decompressor finds that the entry specified by the index associated from the compressed data is empty. This is also inconsistent. These are detected as the exception events and the decompressor regards the compressed data as a command set from the compressor. This is useful function to exchange exceptional commands via a communication data path equipped between the compressor and the decompressor where a data stream is transferred continuously without degrading the compression ratio.

### 2.5. Discussion

As presented in the background above, when targeting visually lossless video transmission, ADPCM can become a method to implement an appropriate communication data path with low latency. However, it is not suitable for unstable connection between the encoder and the decoder because the conventional ADPCM uses a fixed number of bits as the encoded output. If we can employ a new mechanism for the adaptive throughput control to ADPCM by changing the encoding bit length, we can stably transfer the video data with a communication buffer between the encoder and the decoder while the image quality is adaptively controlled. Thus, a new ADPCM with variable encoding mechanism will provide improved and more productive performance for imaging applications such as the object detection by machine learning on IoT devices.

In addition to the visually lossless encoding we focus on in the encoding mechanism of ADPCM above, we expect that the output data stream from the encoding includes the similar bit patterns because the predicted offset adaptively adjusts the origin of the difference regarding the original values. This means that the encoded output from ADPCM forms a low entropy data stream. Here, we can consider employing lossless data compression after the visually lossless encoding. Therefore, we can reduce the data stream from ADPCM compressed in M/N time and also the additional lossless data compression will reduce it further. The lossless compression should treat data stream and achieve a low latency for the operation. Thus, we can employ ASE coding that satisfies the conditions.

According to the discussion above, this paper proposes three methods below. First, we propose a novel ADPCM with a variable encoding bit-length mechanism. Second, employing ADPCM, we propose a video transfer system that includes a data flow control and a buffering mechanism for communication. Finally, employing ASE coding as lossless data compression in the system, we aim to compress the visually lossless data further.

## 3. Visually Lossless Data Compression Applying Variable Bit-Length ADPCM

### 3.1. System Modelling

**Figure 3:**
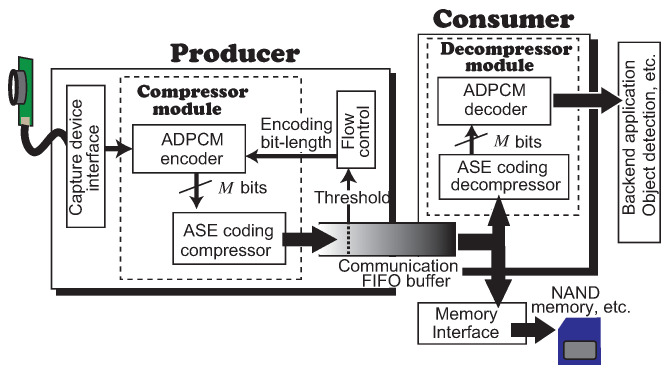
The system model for the visually lossless video transfer.

Let us begin by defining the model of the video transfer system illustrated in Figure 3. The producer is a generator for compressed data stream of video frames from capturing devices such as CMOS image sensors or CCD cameras. The consumer is an application server that decompresses and decodes the received data stream from the producer. Those are connected via an unstable communication media such as Ethernet. We model the path as a First In First Out (FIFO) buffer. FIFO is prepared for sending/receiving the compressed data stream. The producer writes the compressed frame data to the FIFO buffer by applying a variable bit-length encoding depending on a flow control regarding the communication data path. In this paper, we develop an autonomous system that decides whether the encoding bit length is increased/decreased as upgrading/degrading the image quality by applying a new ADPCM.

The producer is organized with a compressor module, a communication FIFO and a flow control module. The compressor module includes the ADPCM encoder that is able to change the bit length *M* and ASE coding’s compressor. The compressed data stream from the compressor module is written to FIFO. ADPCM has an option input that dynamically changes the configured encoding bit length from the flow control module. The flow control is managed by the feedback whether the amount of buffering data stored in FIFO passes a threshold or not. If it passes the threshold, the flow control module generates a signal to ADPCM to decrease the encoding bit length *M*. This reduces the throughput and avoids overflow. As the amount of stored data in FIFO decreases to a lower level than the threshold, the flow control module generates a signal to increase *M*. The output data stream from ADPCM is sent to ASE coding, and, then, it is compressed and written to the FIFO buffer. As explained above, in the producer, the frame data from the capture device will be sent to ADPCM with the mechanism of variable bit length. The encoded data are compressed by lossless data compression. As the bit length is controlled by the threshold of the FIFO buffer, the throughput is adjusted autonomously to the dynamic communication performance.

The consumer is organized with a decompressor module and the communication FIFO buffer. The consumer can decompress and decode the received data from FIFO continuously without any stall by using the decoder of the decompressor of ASE coding and the variable bit-length ADPCM, respectively. Here, the output from the consumer is a pixel data stream. Thus, the video data stream from the producer to the consumer is transferred continuously by controlling the throughput of the communication data path.

Here, let us focus on the mechanism to convey the modification command of the encoding bit length *M* from the producer to the consumer. There are tree methods: (1) a separated communication channel for the command from the data one is prepared; (2) data packeting is performed before writing to FIFO; and (3) the exception symbol is sent from the producer’s ASE coding. The first method has difficulty synchronizing the timings between when the data and the command are received by the consumer. The second method has to implement a protocol for the packeting and increase additional data size for the packet envelope. Therefore, we use the last method because the producer inserts an exception symbol easily in the data stream and the consumer is able to synchronize to change the bit length as soon as it receives the symbol.

In the video transfer system mentioned above, the key technology is ADPCM with a bit-length control. It needs not only a control method to change the encoding bit length by the configuration input but also a method to preserve a visually lossless encoding mechanism in high-quality images. In the next section, we propose the variable bit-length ADPCM.

### 3.2. ADPCM with Variable Bit-Length Control

First, let us consider how we apply an image data stream to the variable bit-length ADPCM encoding. We assume a data stream of each color element in a pixel, such as R, G and B or Y, U and V, is encoded by ADPCM. For example, when the color format is 24 bit RGB, each color element is 8 bit. In this case, each element inputted to the encoder is organized as N=8. Another example is YUV420 [39]. If Y is organized with 8 bit, U and V are both 8 bit while the data size is one fourth of Y because U and V elements are stored every 2×2 pixels. These cases prepare individual encoders/decoders for the streams, respectively. Additionally, the encoder must be controlled by following the frame size of the width and height. We especially need to take care of the last pixel on each line and need to control the encoding to eliminate the effect from the last pixel to the first pixel on the next line. We can reset the encoder and decoder every line by initializing the predicted offset. However, it is not enough because the initial value of the predicted offset may have a large disparity of the color around the first pixel. Therefore, ADPCM needs to have an input to initialize the previous data $P_{i-1}$ and the initial predicted offset *w*.

**Figure 4:**
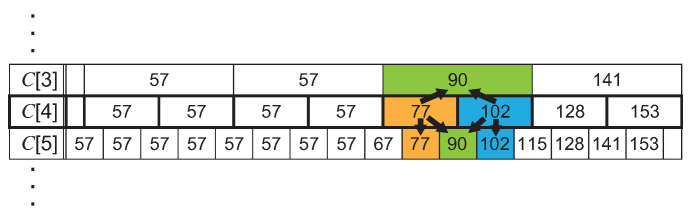
An example of the array *C* in ADPCM-VBL when N=8.

Considering the controls explained above regarding the encoder and the decoder, we propose ADPCM with variable bit-length control (ADPCM-VBL) that extends the interfaces to accept the variable *M* and the specialization for image encoding/decoding into Algorithms 3, 4, 5 and 6. Two parts are affected by the extension. One is the array *C* used in the update for the predicted offset *w*. We need to prepare all available contents in *C* that can be used for any *M*. Here, when the *M* is large enough, the encoded ADPCM value becomes fine grained. This requires that the predicted offset should be fine grained. To support this characteristic, we employ the *C* that includes available contents for any *M*. The initialization of a two-dimensional array *C* is defined in Algorithm 3. The first step of the initialization defines $c_{k}$ where M=N/2 and $0\leq k\leq 2^{M-1}$, as shown in Figure 4, as well as the *C* in Algorithm 1. The contents of *C* where M<N/2 are defined by average values of two neighbor elements in C[M+1] (i.e., (C[M][k]+C[M][k+1])/2). Where M>N/2, the middle value between C[M][k−1] and C[M][k+1] is assigned to C[M][k]. Here, we do not need to consider the case when N=M because it is just a passthrough mode of the input data stream to the output of the encoder/decoder. Thus, the number of arrays in *C* is N−1 and each array includes $2^{M-1}$. The total number of elements in *C* is derived from

$$\sum_{i=1}^{N-1}2^{i-1}=2^{N-1}-1.$$

Another modification from the original ADPCM is the update operation of the predicted offset. As discussed in the previous section, we need to care for the predicted offset and the previous original data to encode/decode the first data in a line of an image. Therefore, we prepare initialization inputs for those values in ADPCM-VBL. Additionally, we need to consider the predicted offset when *M* is changed dynamically depending on the difference between the last *M* and the new *M*. When the difference is positive, the predicted offset should be increased because the resolution of the ADPCM value presented by *M* increases. Otherwise, the offset should be decreased. We use bit shift operation to modify the predicted offset *w* using the difference of *M*. This mechanism provides adaptive encoding/decoding following the dynamic change of *M*.
**Algorithm 3** Initialize the array of the predicted offsets.**Require:***M* // the number of bits for ADPCM encoder. **Require:**
$c_{k}$ // the array of the initial values for C[N/4]. **Ensure:**
*C* // the array used for the predicted offset amount. // When M=N/2 **for**
j=0 to $2^{N/2-1}$ **do**  
$C\left[N/2\right]\left[j\right]\leftarrow c_{k}$ **end for** // When M<N/2 **for**
i=N/2−1 downto 1 **do**  **for**j=1 to $2^{i-1}$ **do**   
C[i][j−1]←(C[i+1][j×2−2]+C[i+1][j×2−1])/2  **end for** **end for** // When M>N/2 **for**
i=N/2+1 to *N* **do**  
C[i][0]←C[i−1][0]  
j←2  **while**
$j<2^{i-1}$
**do**   
C[i][j−1]←C[i−1][j/2−1]   
C[i][j]←(C[i−1][j/2−1]+C[i−1][j/2])/2   
j←j+2  **end while**  
$C\left[i\right]\left[2^{i-1}-1\right]\leftarrow C\left[i-1\right]\left[j/2-1\right]$ **end for**


The algorithms of initialization and encoding/decoding for ADPCM-VBL that the modifications above are applied are shown in Algorithms 4–6, respectively. Before encoding/decoding using those algorithms, the initialization is invoked when the video transfer system is reset. The encoding function has argument inputs for *w* and $P_{prev}$. Those are given by the video transfer system. When the input value for *M* is modified, the predicted offset *w* is shifted by the number of bits in the difference between *M* and $M_{prev}$. According to the algorithms, ADPCM-VBL will encode the inputted data stream into *M* bit ADPCM value following the changes of the original data. Now, we apply the algorithms to the video transfer system.
**Algorithm 4** Initialization for variable bit-length ADPCM.**Require:***N* // N bit Pixel element. **Require:**
C_INIT[ ] // N bit Pixel element. 
w←
W_INIT 
$P_{prev}\leftarrow$
P_INIT 
$f_{prev}\leftarrow w$ 
$M_{curr}\leftarrow$
M_INIT


**Algorithm 5** Variable bit-length ADPCM encoder.**Require:***P* // N bit Pixel element. **Require:**
*M* // bits of the current ADPCM value. **Require:**
InitW // if the predicted offset is initialized or not. **Require:**
$W_{init}$ // a predicted offset to be initialized. **Require:**
InitP // if the $P_{prev}$ is initialized or not. **Require:**
$P_{init}$ // an original data to be set to $P_{prev}$. **Ensure:**
*p* // ADPCM value. **if**
M=N
**then**  
p←P  **return** **end if** **if**
$M\neq M_{prev}$
**then**  **if**
$M>M_{prev}$
**then**   
$w\leftarrow w\ll (M-M_{prev})$  **else**   
$w\leftarrow w\gg (M_{prev}-M)$  **end if** **end if** **if**
InitW=true
**then**  
$w\leftarrow W_{init}$ **end if** **if**
InitP=true
**then**  
$P_{prev}\leftarrow P_{init}$ **end if** 
$d\leftarrow P-P_{prev}$ 
$step\leftarrow \left(w\times 2\right)/2^{M-1}$ **if**
d>0
**then**  **if**
$\left|d\right|/step>2^{M-1}-1$
**then**   
$p\leftarrow 2^{M-1}-1$  **else**   
p←ceil(d/step)  **end if** **else**  **if**
$\left|d\right|/step>2^{M}-1$
**then**   
$p\leftarrow 2^{M}-1$  **else**   
$p\leftarrow ceil\left(-d/step\right)+2^{M-1}$  **end if** **end if** 
$f_{prev}\leftarrow f_{prev}\times C\left[p\&\left(\left(1\ll M\right)-1\right)\right]/64$ 
$w\leftarrow f_{prev}$


**Algorithm 6** Variable bit-length ADPCM decoder.**Require:***p* // ADPCM value. **Require:**
*M* // bits of the current ADPCM value. **Require:**
InitW // if the predicted offset is initialized or not. **Require:**
$W_{init}$ // a predicted offset to be initialized. **Require:**
InitP // if the $P_{prev}$ is initialized or not. **Require:**
$P_{init}$ // an original data to be set to $P_{prev}$. **Ensure:**
*P* // N bit Pixel element. **if**
M=N
**then**  
P←p  **return** **end if** **if**
$M\neq M_{prev}$
**then**  **if**
$M>M_{prev}$
**then**   
$w\leftarrow w\ll (M-M_{prev})$  **else**   
$w\leftarrow w\gg (M_{prev}-M)$  **end if** **end if** **if**
InitW=true
**then**  
$w\leftarrow W_{init}$ **end if** **if**
InitP=true
**then**  
$P_{prev}\leftarrow P_{init}$ **end if** 
$step\leftarrow \left(w\times 2\right)/2^{M-1}$ **if**
$p<2^{M-1}$
**then**  
d←step×(p×2+1)/2 **else**  
$d\leftarrow -step\times (\left(p-2^{M-1}\right)\times 2+1)/2$ **end if** 
$P\leftarrow P_{prev}+d$ 
$f_{prev}\leftarrow f_{prev}\times C\left[p\&\left(\left(1\ll M\right)-1\right)\right]/64$ 
$w\leftarrow f_{prev}$


### 3.3. Application Examples with Variable Bit-Length ADPCM

Here, let us propose video transfer applications with ADPCM-VBL. As illustrated in Figure 3, we can consider two types of applications. One is an application that has a communication data path connecting the producer and the consumer. This is typically a network application with unstable throughput that transfers video data to a server, and the server analyzes the video images. Another is the one with only the producer. The output is saved in a local storage such as NAND-based memory card. This application has the advantage of avoiding a limitation for the speed on write operation that depends on manufacturing technology of the memory.

Now, we focus on the former type of applications with the producer and consumer as well as the one equivalent to the video transfer system mentioned in Section 3.1. The latter can be implemented by the producer part of the former one. The producer and the consumer of this application are controlled by Algorithms 7 and 8, respectively. During the system reset, the initialization of Algorithm 4 is invoked. The producer compresses data blocks from the capturing device input. The data block is organized with one or more pixel elements. The producer receives $M_{curr}$ that is the input to change the encoding bit length of ADPCM. *FifoTh* is a constant value of the threshold in the communication FIFO buffer. If the amount after a data block is written in FIFO becomes more than the threshold, $M_{curr}$ is decremented. Otherwise, it is incremented to increase the image quality. When the increment/decrement occur, ASE coding is reset, and the input symbol width is modified according to the new $M_{curr}$. The symbol width for ASE coding should be large enough to achieve good compression ratio. We define the symbol width as $S_{w}\times M_{curr}$ where $S_{w}$ is an integer greater than 0. The predicted offset and the previous color data $P_{prev}$ is reset every number of pixels in a line. The line width is defined by a constant value WIDTH. The color element of the first pixel in a line is saved to $P_{init}$ and used for the initialization.

The data block size mentioned above is an integer value greater than 0. During the encoding/decoding the data block, $M_{curr}$ is not modified regardless of the threshold detection of FIFO. Therefore, the symbol width is neither changed. The smaller the data block size is, the lower the latency the stream-based encoding/decoding achieves. However, note that too small a data block is not effective for ASE coding because every modification of $M_{curr}$ causes a reset to ASE coding. If it is too big, $M_{curr}$ does not change for a long time. This brings degradation of image quality while $M_{curr}$ is a small value. Thus, we need to decide a suitable size of the data block.
**Algorithm 7** Producer function.**Require:**SendBlockSize **Require:**Block[] **Require:**$M_{curr}$ **Ensure:**BlockSizeC **Ensure:**BlockC  
FullCheck←AFifoCurrSize+SendBlockSize **if**
FullCheck≤FifoTh
**then**  
$M_{curr}\leftarrow M_{curr}<N?M_{curr}+1:N$ **else**  
$M_{curr}\leftarrow M_{curr}>1?M_{curr}-1:1$ **end if** 
i←0 **while**
SendBlockSize>i
**do**  **if**
WidthCount=0
**then**  
$BlockP\left[i\right]\leftarrow AdpcmEncoder(Block\left[i\right],M_{curr},$                                                  $true,W_{init},$                                                  $true,P_{init})$  
WidthCount←
WIDTH **else**  
$BlockP\left[i\right]\leftarrow AdpcmEncoder(Block\left[i\right],M_{curr},$                                                  false,null,                                                  false,null) **end if** **if**
WidthCount=
WIDTH
**then**  
$P_{init}\leftarrow Block\left[i\right]$ **end if** 
i←i+1 
WidthCount←WidthCount−1 **end while** **if**
$M_{prev}\neq M_{curr}$
**then**  
$AseChangeDataWidth(M_{curr})$  
$AseSendException(M_{curr})$  
$M_{prev}\leftarrow M_{curr}$ **end if** 
AseCompression(BlockSize,BlockP,                        ;          
&BlockSizeC,&BlockC)


**Algorithm 8** Consumer function.**Require:**RecvBlockSize **Require:**BlockC **Require:**$M_{curr}$ **Ensure:**BlockSize **Ensure:**Block **if**$M_{prev}\neq M_{curr}$**then**  
$AseChangeDataWidth(M_{curr})$  
$M_{prev}\leftarrow M_{curr}$ **end if** 
AseDecompression(RecvBlockSize,BlockC,                                  
&BlockSize,&BlockP) 
i←0 **while**
BlockSize>i
**do**  **if**
WidthCount=0
**then**   
$BlockP\left[i\right]\leftarrow AdpcmDecoder(BlockP\left[i\right],M_{curr},$                                                  $true,W_{init},$                                                  $true,P_{init})$   
WidthCount← WIDTH  **else**   
$Block\left[i\right]\leftarrow AdpcmDecoder(BlockP\left[i\right],M_{curr},$                                                  false,null,                                                  false,null)  **end if**  **if**
WidthCount= WIDTH **then**   
$P_{init}\leftarrow Block\left[i\right]$  **end if**  
i←i+1  
WidthCount←WidthCount−1 **end while**


The offset of increasing/decreasing $M_{curr}$ relates to a tradeoff between the image quality and the throughput. If the one of decreasing $M_{curr}$ is large, the recovery from the status of FIFO full may become fast. However, the degradation of image quality will become large. Besides, if the one of increasing $M_{curr}$ is small, the number of color data with degradation increases. Here, we use 1 as the offset for increasing/decreasing $M_{curr}$. Of course, we can use unbalanced increasing/decreasing offsets for $M_{curr}$. When $M_{curr}$ is modified, an exception symbol is generated by ASE coding, written to the FIFO buffer, and then the event is conveyed to the consumer.

The consumer’s algorithm in the application is shown in Algorithm 8. After the consumer receives an exception symbol by ASE coding, $M_{curr}$ in ADPCM-VBL is modified and the subsequent uncompressed data from ASE coding is decoded by ADPCM-VBL without any flow control. The consumer performs the same operations regarding the predicted offset and the previous pixel data at the beginning of a line in an image frame as the producer.

In summary, as proposed in this section, we improve the conventional ADPCM to accept dynamic modification of the bit length in ADPCM for encoding/decoding operations. It seamlessly adopts ADPCM to applications that support visually lossless compression. Additionally, we combine lossless data compression after the encoding by ADPCM-VBL. This is expected to further compress the encoded data. We propose a model of a producer and a consumer connected via a communication data path applying the encoding/decoding mechanisms. The system transfers the captured image in a stream, and the throughput is adaptively controlled by the variable bit length in ADPCM. We expect that the quality of image also follows the color changes fluently. We evaluate the proposed methods above in the aspect of the compression performance and the image quality in the next section.

## 4. Experimental Evaluation

### 4.1. Experimental Setup

**Figure 5:**
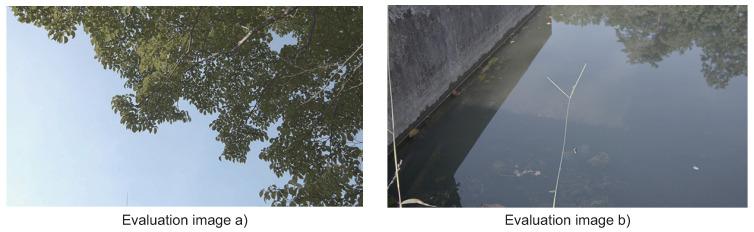
The first frames of two videos used for evaluations.

We evaluated ADPCM-VBL and the system employing it. We focused on the performance aspects of the compression ratios and the image quality. We performed two evaluations: (1) the evaluation of ADPCM-VBL; and (2) the evaluation of the system presented in Section 3.3. We used two videos stored in YUV420 format that were captured by the Blackmagic Pocket Cinema Camera 6K and saved in BRAW format. The first frames are shown in Figure 5. The resolutions of the images are both 3840 × 2160. The data size per YUV420 frame is 12 Mbyte. We gathered the same elements in the order of Y, U and V elements (i.e., the file includes the Y part, U part and V part in the order). Here, Y, U and V are all 8 bit.

The constant parameters for encoder/decoder of ADPCM-VBL used in Algorithm 4–6 are defined by $W_{init}=2^{M_{init}-1}$, $P_{init}=2^{N-1}$ and $M_{init}=N$, where *N* is the bit length of the original data. Note that, during the evaluations below, N=8 due to the YUV420 format. Thus, we used heuristically $W_{init}=128$, $P_{init}=128$ and $M_{init}=8$ in the experiments. However, the evaluation results disseminated in this section do not differ largely even if we applied other settings because those values were fitted to adaptive ones during the encoding/decoding. On the other hand, regarding the settings of ASE coding, we configured to perform the entropy culling at every two hits and implemented 256 entries in the look-up table. We set $S_{w}$ to 4 as the multiplied number with *M* for the symbol width in ASE coding, as mentioned in Section 3.3.

During the evaluations, we used two metrics for the image quality and the compression ratio. The first is Peak Signal-to-Noise Ratio (PSNR) [40]. A larger PSNR (dB) represents equality compared to the original image with respect to each pixel from the Mean Squared Errors (MSE) as follows;

$$MSE=\frac{1}{N}\sum_{i=0}^{N}{\left\{P_{o}\left(i\right)-P_{e}\left(i\right)\right\}}^{2}$$

where *N*, $P_{o}$ and $P_{e}$ are the number of pixels in a frame, the original pixel value and the encoded one. The PSNR of a frame is calculated by the following equation;

$$PSNR=20\log_{10}\frac{MAX}{\sqrt{MSE}}.$$

MAX is the maximal number of a pixel value. According to the literature [41], the values of several videos encoded by H.264 vary within 31–38. This means around 35 (dB) preserves high visual quality equivalent to an 800 kbps video stream. The second is presented by the percentage derived from: (compressed data size/original data size) × 100. Depending on evaluations, the original/compressed data are the ones of the input/output from ADPCM-VBL or the input data to ADPCM-VBL or from ASE coding. We explain the combination according to the objective in each evaluation.

### 4.2. Evaluation for Variable Bit-Length ADPCM Encoding

Let us begin from the evaluation of ADPCM-VBL focusing on image quality of encoding and the compression performance. We performed the encoding/decoding of the frames shown in Figure 5 by varying the encoding bit length *M*. We evaluated the image quality according to the PSNRs of the decoded images. We also evaluated the compression ratios derived from ASE coding where the encoded data stream generated by ADPCM-VBL is inputted.

**Figure 6:**
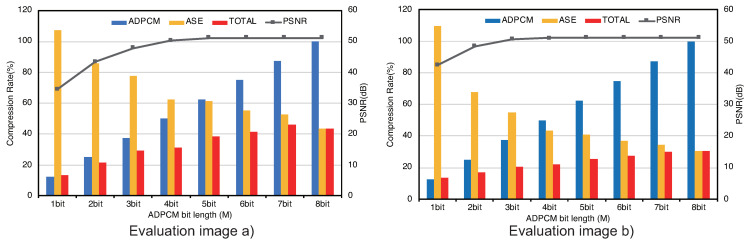
PSNRs and compression ratios regarding ADPCM-VBL and ASE coding. The graph in the left and the one in the right show the results regarding the evaluation image a) and b) shown in Figure 5, respectively.

**Figure 7:**
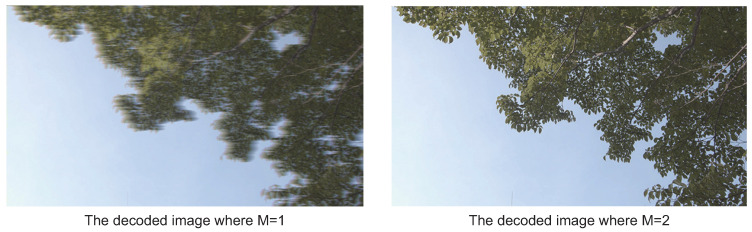
The decoded images when M=1 and M=2 using the evaluation image a) shown in Figure 5.

**Figure 8:**
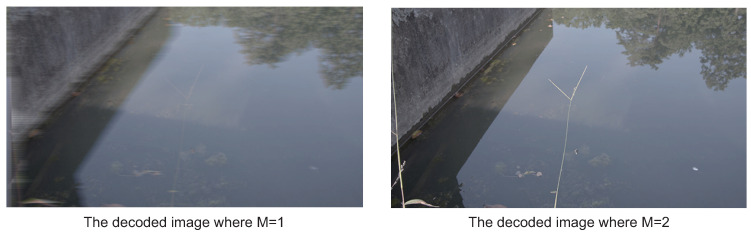
The decoded images when M=1 and M=2 using the evaluation image b) shown in Figure 5.

The graphs depicted in Figure 6 show the PSNRs after decoding by a line and the compression ratios by bars. The vertical axis on the left side is the compression ratio. The one on the right side is the PSNR. Regarding Evaluation Image (a), the PSNRs when varying the encoding bit length *M* from 4 to 8 show about 50. As *M* is decreasing from 3 to 1, the PSNRs are decreasing. However, we confirmed any *M* maintains adequate image quality because all PSNRs in this experiment show values greater than 30. Here, let us focus on the visual qualities between the bit lengths *M* of 1 and 2, as shown in Figure 7. When M=1, as shown on the left in Figure 7, the image becomes blurry in the horizontal direction. This is caused by the low flexibility because the predicted offset is decided by only a single bit. When M=2, as shown on the right in Figure 7, it is sharper than the image of M=1. However, it still has blurry parts in edges of colors such as the one between a leaf and the sky in the image. This is a typical encoding characteristic because ADPCM cannot follow such large changes of color values when the encoding bit length is small. On the other hand, Evaluation Image (b) also maintains the same relationship as Evaluation Image (a) between the image quality and *M*, as shown in Figure 8. Thus, we confirmed that we should avoid M=1 to maintain adequate image quality as well as restrict the condition 2≤M≤N in applications of ADPCM-VBL.

Next, let us evaluate the compression performance. The bar graphs in Figure 6 show three types of compression ratios: “ADPCM” shows the ratios after encoding the original data by ADPCM-VBL; “ASE” shows the ones after compressing the encoded data by ASE coding; and “TOTAL” shows the total compression ratio with ADPCM-VBL and ASE coding. For example, in the case of Evaluation Image (a) with M=2, the encoder compresses the original frame data into 2/8×100=25% and ASE coding compresses the encoded data into 85.61%. Finally, the total compression ratio becomes 25/85.61×100 = 21.40%. According to the bar graphs, we confirmed that the lossless compression is effective while *M* is varying from 2 to 8 regarding both evaluation images. Especially, when M=2, the compression ratio becomes about 20% in both cases.

According to the evaluations presented above, we confirmed that ADPCM-VBL can achieve adequate visual quality to the image frames. Especially, even if the encoding bit length is small such as M=2, we confirmed that the degradation of the image quality is low. However, when M=1, the degradation becomes large and the image has significant blur. Therefore, the case is not acceptable for visually lossless compression. Furthermore, we confirmed that the lossless data compression works effectively after the encoding by ADPCM. This proves the hypothesis discussed in Section 2.5 that we expected low entropy in the encoded data generated from ADPCM. Thus, we concluded that it is an effective combination that the video data stream is encoded by ADPCM-VBL and compressed by ASE coding.

### 4.3. Evaluation for Video Transfer System with ADPCM-VBL

**Figure 9:**
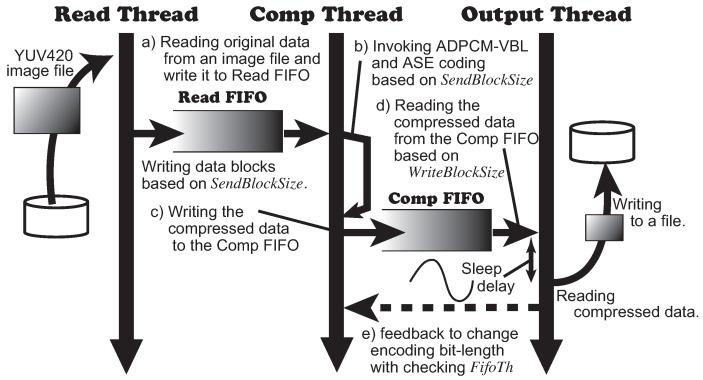
The organization and the data flow of the emulator.

Next, we evaluate the application with the communication data path explained in Section 3.3 using the algorithms for the producer. We focus on the validities of the dynamic control of the communication throughput and the quality of the images. In this evaluation, we implemented a software emulator with the producer using multiple threads. Those threads read video data from file, encode/compress the data and write the compressed data into another file. The execution environment was organized on a Windows 10 machine with Intel Core i5 3.3 GHz and 24 GB memory. Figure 9 illustrates the organization and the dataflow of the emulator. There are three threads in the emulator. The *Read Thread* reads video data from a file and writes it to *Read FIFO* buffer (Figure 9a). The *Comp Thread* implements Algorithm 7 that performs ADPCM-VBL and ASE coding (Figure 9b) and then writes the compressed data to the *Comp FIFO* (Figure 9c). The Comp Thread works at every writing an amount *SendBlockSize* of video data into the Read FIFO. Finally, the *Output Thread* reads the compressed data from the Comp FIFO and writes it into a file (Figure 9d). The Output Thread works at every writing an amount *WriteBlockSize* of video data into the Comp FIFO. The Output Thread checks the threshold *FifoTh* of the Comp FIFO and informs if the data size in FIFO passes the threshold or not (Figure 9e). Here, the Output Thread emulates the communication overhead with inserting a delay while the thread is writing the compressed data to a file. We implemented the delay using the *sleep* function in the host system.

We configured the parameters in the emulator and Algorithm 7 as follows: SendBlockSize is four pixels (i.e., four bytes due to N=8), WriteBlockSize is 4096 byte and *FifoTh* is WriteBlockSize × 200 byte. We used two contiguous frames of the evaluation video that the first frame is shown as the evaluation image a) in Figure 5. According to the result in the previous section, we applied 2≤M≤N. The delay was generated periodically by a sine function and passed as the argument of the sleep function.

Applying the emulator, we focused on the following three evaluation points. First, observing the Comp FIFO, we evaluated the performance for the throughput control by ADPCM-VBL in the communication data path. Second, we evaluated the realtime compression ratios during the emulation by comparing the data amount written into the Read FIFO and the one read from the Comp FIFO. Finally, we evaluated the image qualities of the transferred image frames.

**Figure 10:**
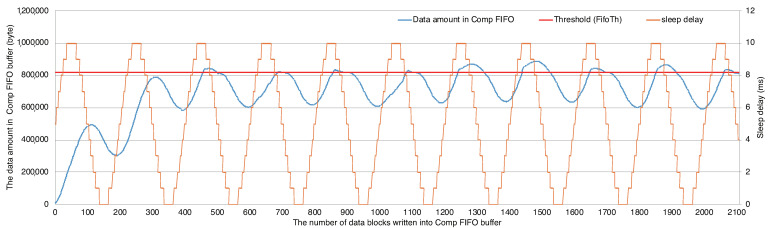
The data amount maintained in Comp FIFO and the communication overhead (delay) given during the emulation.

Figure 10 shows the realtime data amount of the Comp FIFO in the left axis with the blue line and the delay in the right axis with the orange line. The horizontal axis is the number of WriteBlockSize written to the Comp FIFO. The threshold *FifoTh* of FIFO is depicted by the red horizontal line. The frequency of the delay given by sine function is 100 ms. The amplitude is 5 ms. As the number of the data blocks written to FIFO is increased, the amount of data in the FIFO buffer reaches the threshold. For example, although the data amount passes the threshold around the 500th data block, it is reduced soon due to the feedback control by the producer and the autonomous control of the encoding bit length by ADPCM-VBL. Thus, we confirmed that the producer adaptively controls the throughput with the dynamic compression mechanism.

**Figure 11:**
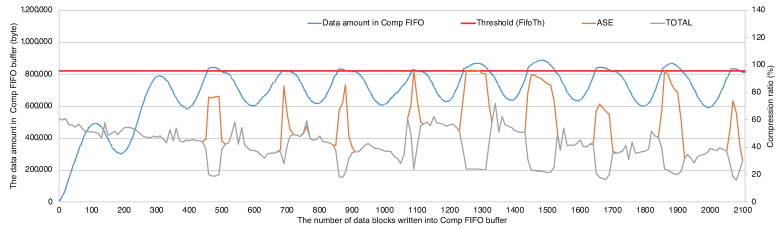
The data amount maintained in Comp FIFO and the compression ratios during the emulation.

**Figure 12:**
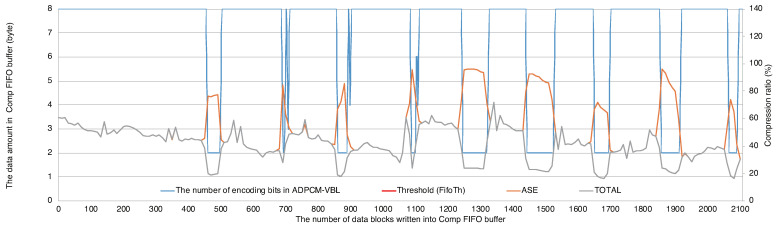
The number of encoding bits in ADPCM-VBL (M) and the compression ratios during the emulation.

Next, let us evaluate the compression ratios referring two graphs. Figure 11 depicts the compression ratios of every data block written to the Comp FIFO during the emulation with respect to the right axis. The gray line shows the total compression ratio after ADPCM-VBL and ASE coding. The orange line shows the one only by ASE coding. Figure 12 shows the number of bits *M* used for ADPCM encoding in the left axis with blue line. When the data amount in FIFO does not pass the threshold, the producer keeps M=8 and passthroughs the original pixel data. In this case, the total compression ratio becomes equivalent to that of ASE coding. As *M* is decreased when the data amount in FIFO passes the threshold, the producer tries to reduce the data amount in FIFO below the threshold. According to Algorithm 7, the compression ratio becomes bad instantly due to the reset of ASE coding right after *M* is changed. However, we confirm that ASE coding achieves effective compression ratios at almost all situations. The average compression ratio of ASE coding during this emulation becomes 48.25%. This shows lossless data compression works effectively against the encoded data from ADPCM-VBL.

We discuss the effect of the lossless data compression after ADPCM-VBL encoding focusing on the theoretical aspect of ASE coding. The compression ratio is derived by the following equation according to our previous work [20]:

$$\frac{(m_{avg}+1)h}{s}+\frac{\left(s+1)(1-h\right)}{s}=0.4825.$$

Here, $m_{avg}$ is an average bit length of the compressed data after the entropy calculation, *s* is the bit length of the original data and *h* is the average hit rate in the look-up table. If h=0, the compression ratio becomes more than 100%. Therefore, *h* must be more than 0.5. Besides, the first term of the equation above must be less than 0.5. Assuming that the worst case of the hit ratio is 0.5, we derive the relationship below:

$$\frac{\left(m_{avg}+1\right)}{s}+\frac{\left(s+1\right)}{s}.$$

We find the relation $m_{avg}<s$. This means that ASE coding shrinks an encoded data to a smaller number of bits than the original data size in a short time window restricted by the size of the look-up table in a stream manner. Thus, we confirmed that the encoded data stream by ADPCM-VBL has potentially low entropy and also has the possibility to be compressed.

**Figure 13:**
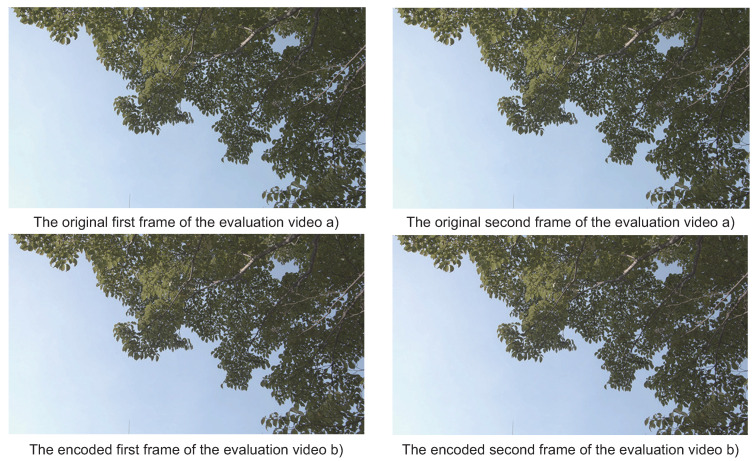
The first and the second frames encoded by the emulation.

Finally, let us evaluate the image quality transferred by this emulation. Figure 13 shows the contiguous image frames used in this experiment before/after the compression. The variable bit-length control of ADPCM-VBL makes some continuous pixels suddenly blurry due to the bit error propagation when *M* is decremented. However, the degradation is recovered quickly by the adaptive characteristic of the predicted offset. Therefore, the visual image qualities are maintained because the PSNRs of those frames are 46.68 and 44.48, respectively. Regarding the PSNR of the first frame, it is equivalent to the one of M=3 according to the graph in Figure 6. Despite that the minimal *M* is 2, we confirmed that the emulation resulted in better image quality.

According to the evaluations above, we confirmed that it is available to develop an effective and stream-based image transfer system with ADPCM-VBL and ASE coding. We maintain the image quality adequately with changing the bit length used for the encoding from 2 bits to *N* bits. Moreover, we confirmed that the data entropy of the encoded data from ADPCM becomes low. This allows the lossless data compression to reduce the data size further. Thus, we concluded that the evaluations presented in this section proved the efficiencies of ADPCM-VBL and the system with the encoding that combines the lossless compression mechanism.

## 5. Conclusions

We proposed a novel stream-based method of visually lossless data compression that transfers image data in low latency and high quality applying ADPCM. We proposed a new ADPCM that allows dynamically changing the encoding bit length, called ADPCM-VBL. Furthermore, since we expected that the data entropy becomes low after the encoding of ADPCM, we proposed a video transfer system that combines the encoder and a lossless data compressor applying ASE coding. Applying the new algorithms, we evaluated the video transfer system by an emulation in the aspects of the compression ratio and the image quality. We confirmed that ADPCM-VBL effectively reduces the amount of data adaptively against the dynamic throughput changes in the communication data path. Additionally, we confirmed that the lossless data compression after the encoding by ADPCM effectively works to reduce the data amount. Finally, we confirmed that ADPCM-VBL achieves high visual quality, preserving all pixels under the condition that the encoding bit length is more than 2. Thus, we concluded that the methods proposed in this research are proven as effective and worthy for video applications that need fast communication and high visual quality.

For future works, we are planning to implement the proposed methods in hardware. Then, we will apply it to the display application such as HDMI. We will also evaluate the methods to prove the validity in the object detection applications such as CNN-based analysis.

## Data Availability

The image files in the original resolutions resulted from the evaluations are available from the supplemental material. The compressed file includes the files in bitmap format stored in the corresponding directories to the figures in the paper.

## Supplementary Materials

The following are available online at https://www.mdpi.com/article/10.3390/s21134602/s1.
