# Supplementary figures and images for: Stream-Based Visually Lossless Data Compression Applying Variable Bit-Length ADPCM Encoding
Source: Sensors (Basel). 2021 Jul 5;21(13):4602. doi: 10.3390/s21134602 (PMC8271783; doi:10.3390/s21134602)

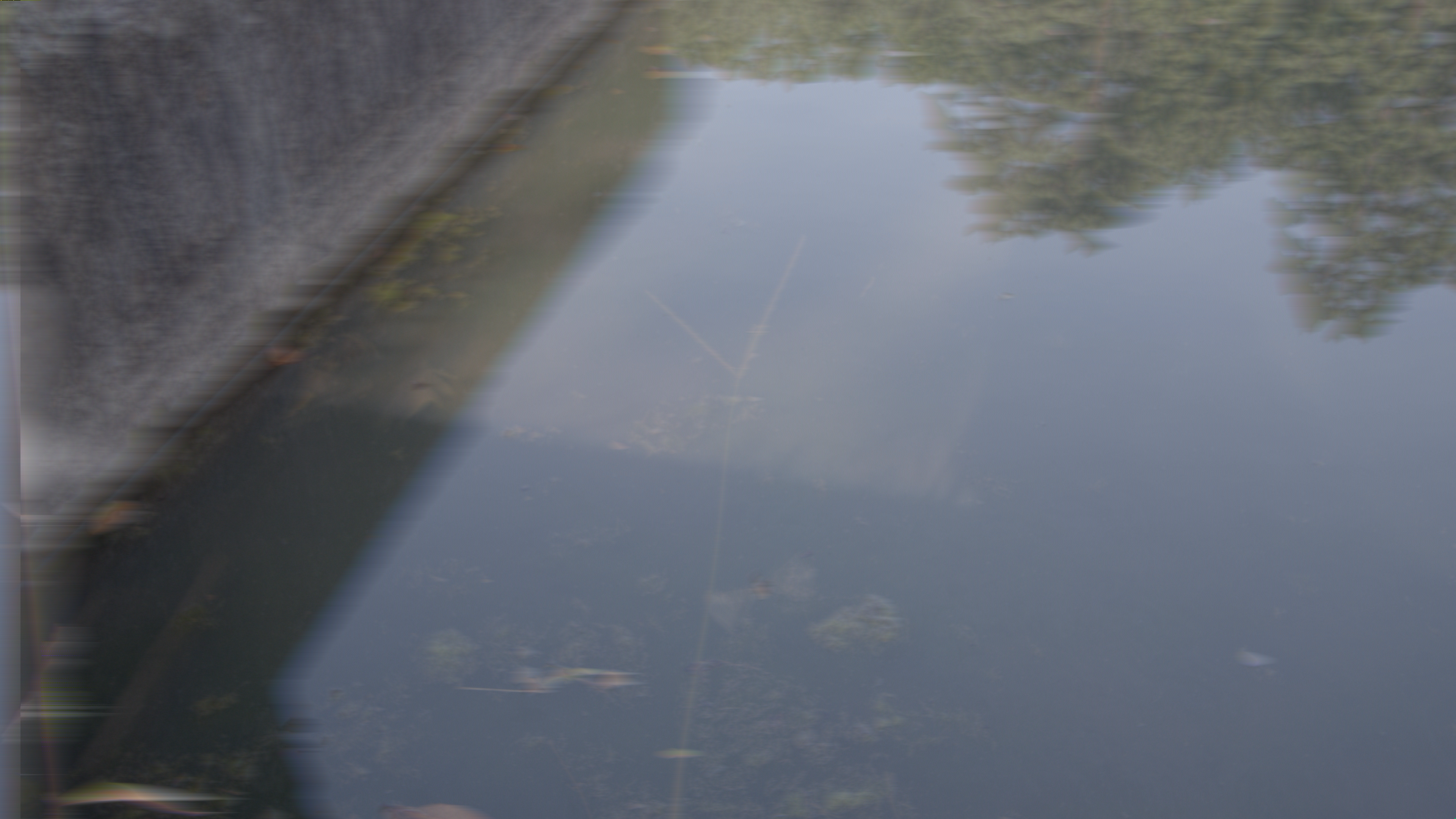

Supplement: Supplementary file 1 [file sensors-21-04602-s001.zip › supplimental_figures/Figure8/Figure8_M_1.bmp]

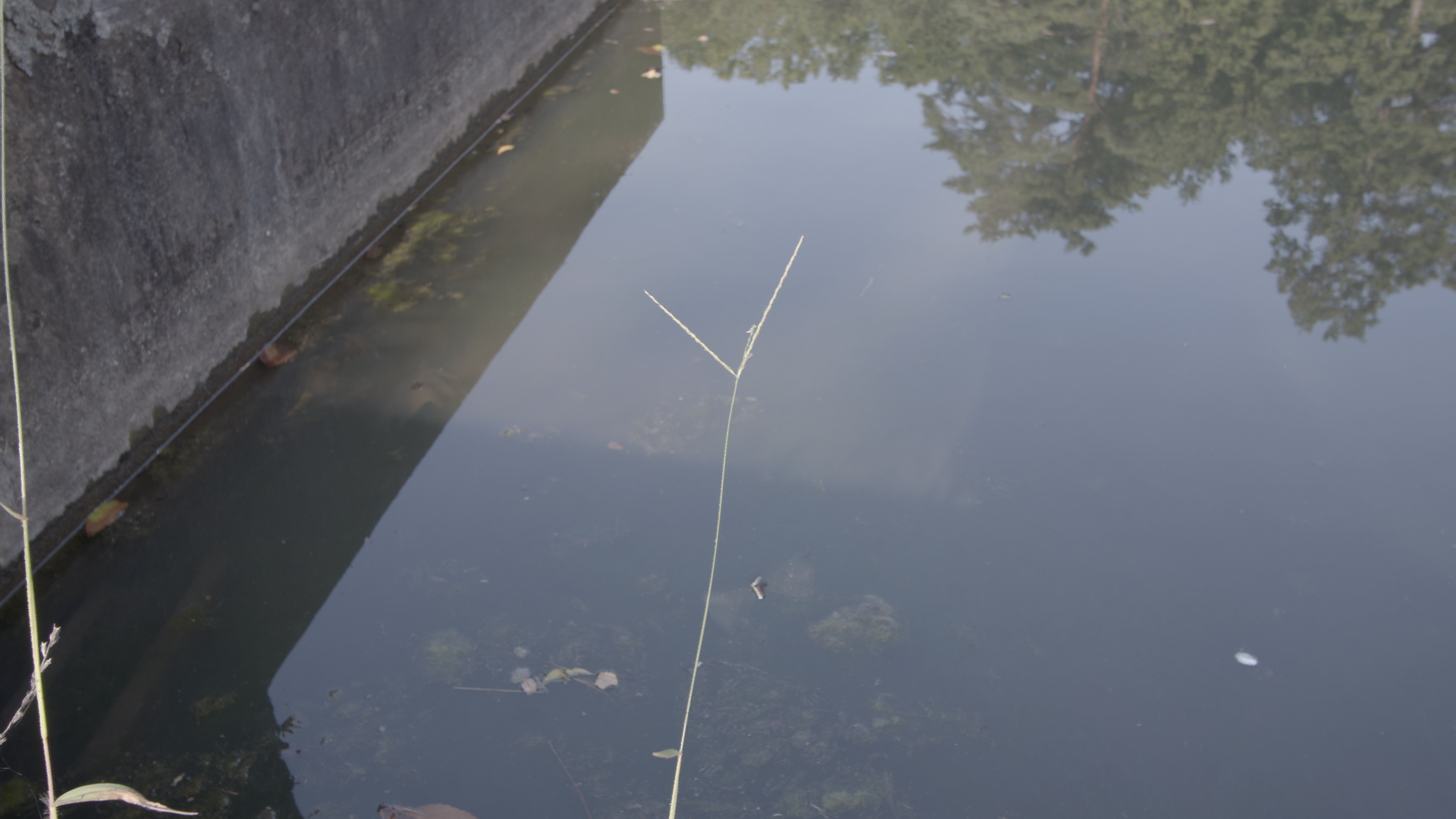

Supplement: Supplementary file 1 [file sensors-21-04602-s001.zip › supplimental_figures/Figure8/Figure8_M_2.bmp]

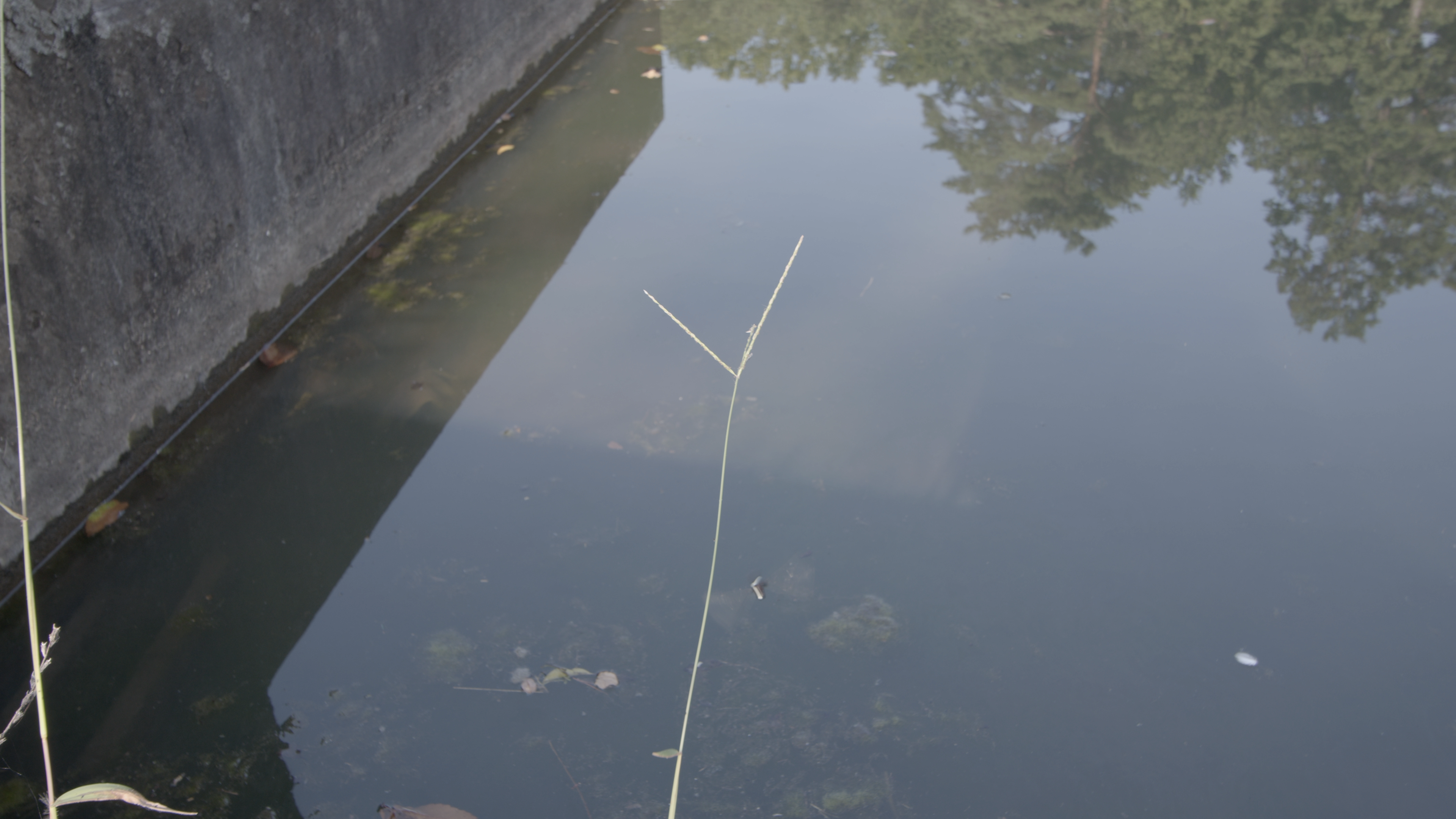

Supplement: Supplementary file 1 [file sensors-21-04602-s001.zip › supplimental_figures/Figure5/Figure5_evaluation_image_b.bmp]

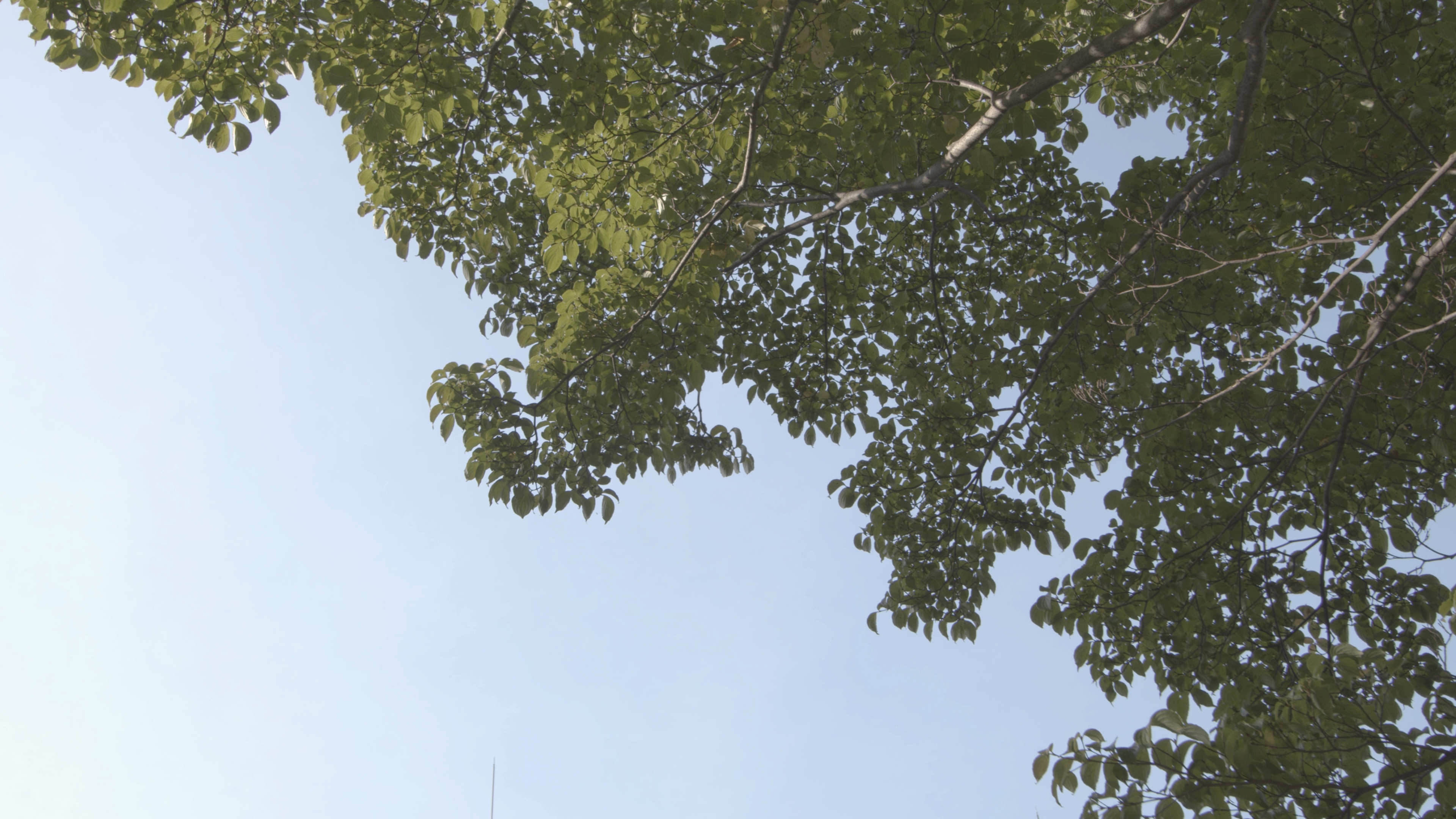

Supplement: Supplementary file 1 [file sensors-21-04602-s001.zip › supplimental_figures/Figure5/Figure5_evaluation_image_a.bmp]

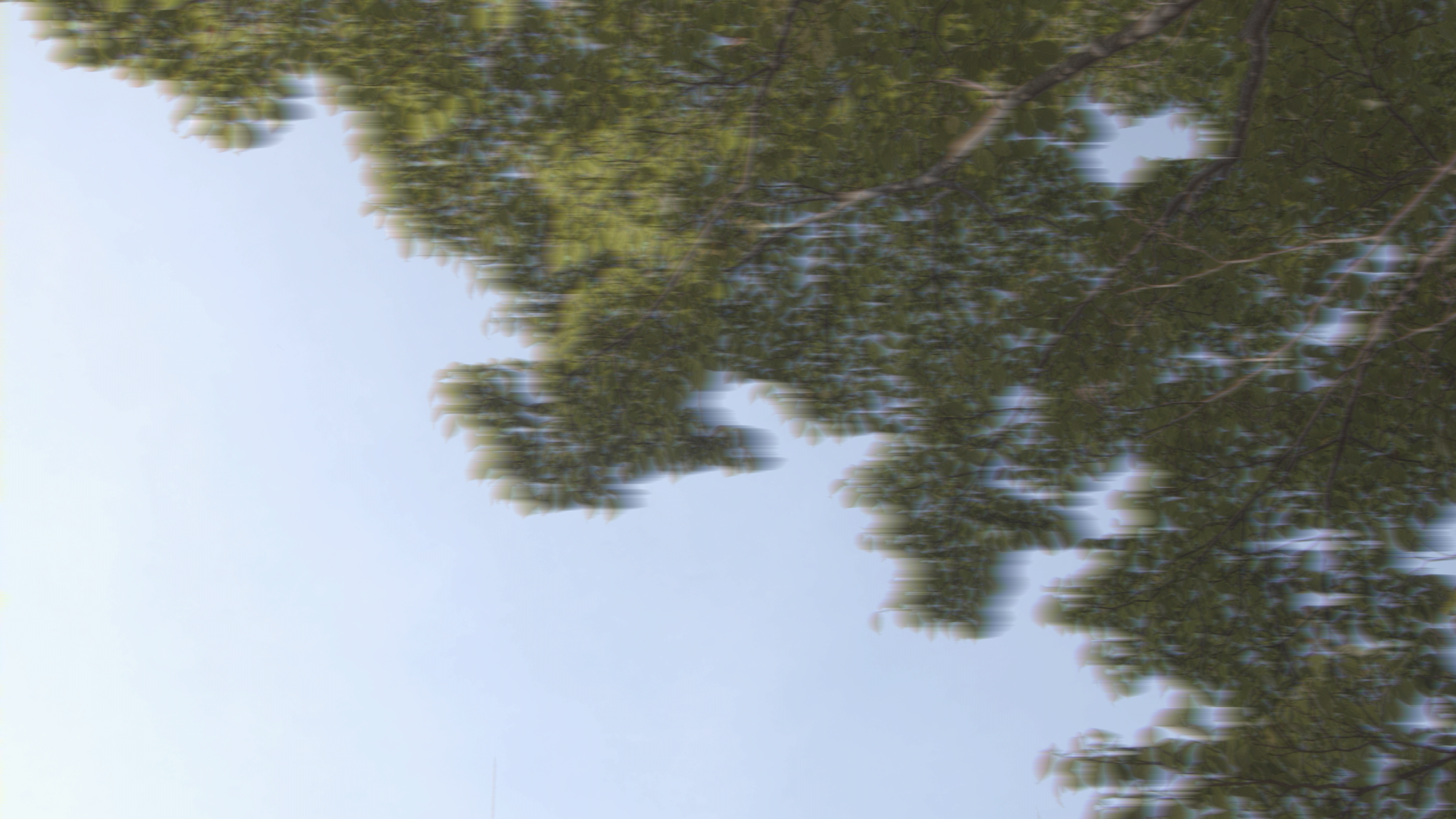

Supplement: Supplementary file 1 [file sensors-21-04602-s001.zip › supplimental_figures/Figure7/Figure7_M_1.bmp]

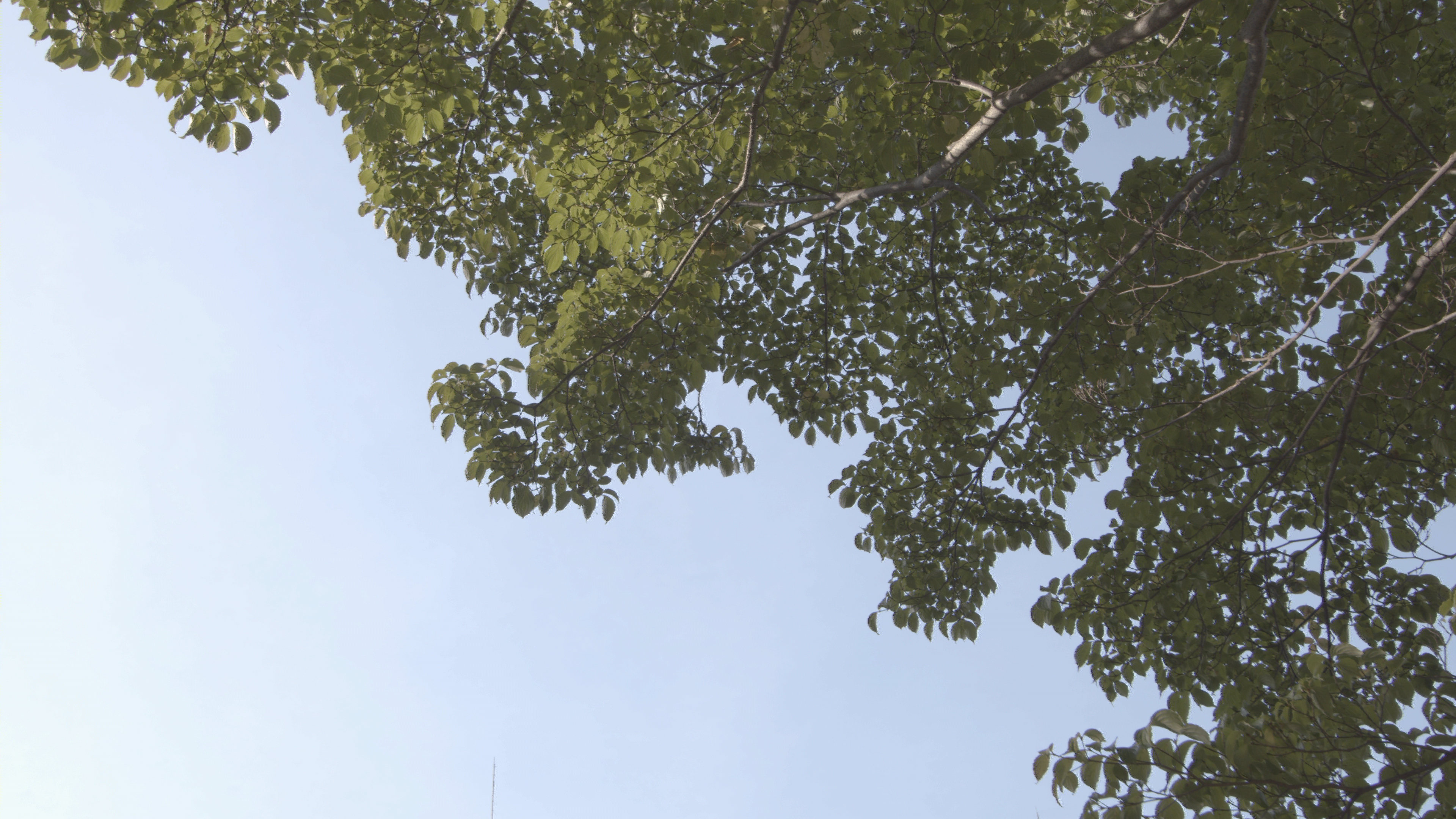

Supplement: Supplementary file 1 [file sensors-21-04602-s001.zip › supplimental_figures/Figure7/Figure7_M_2.bmp]

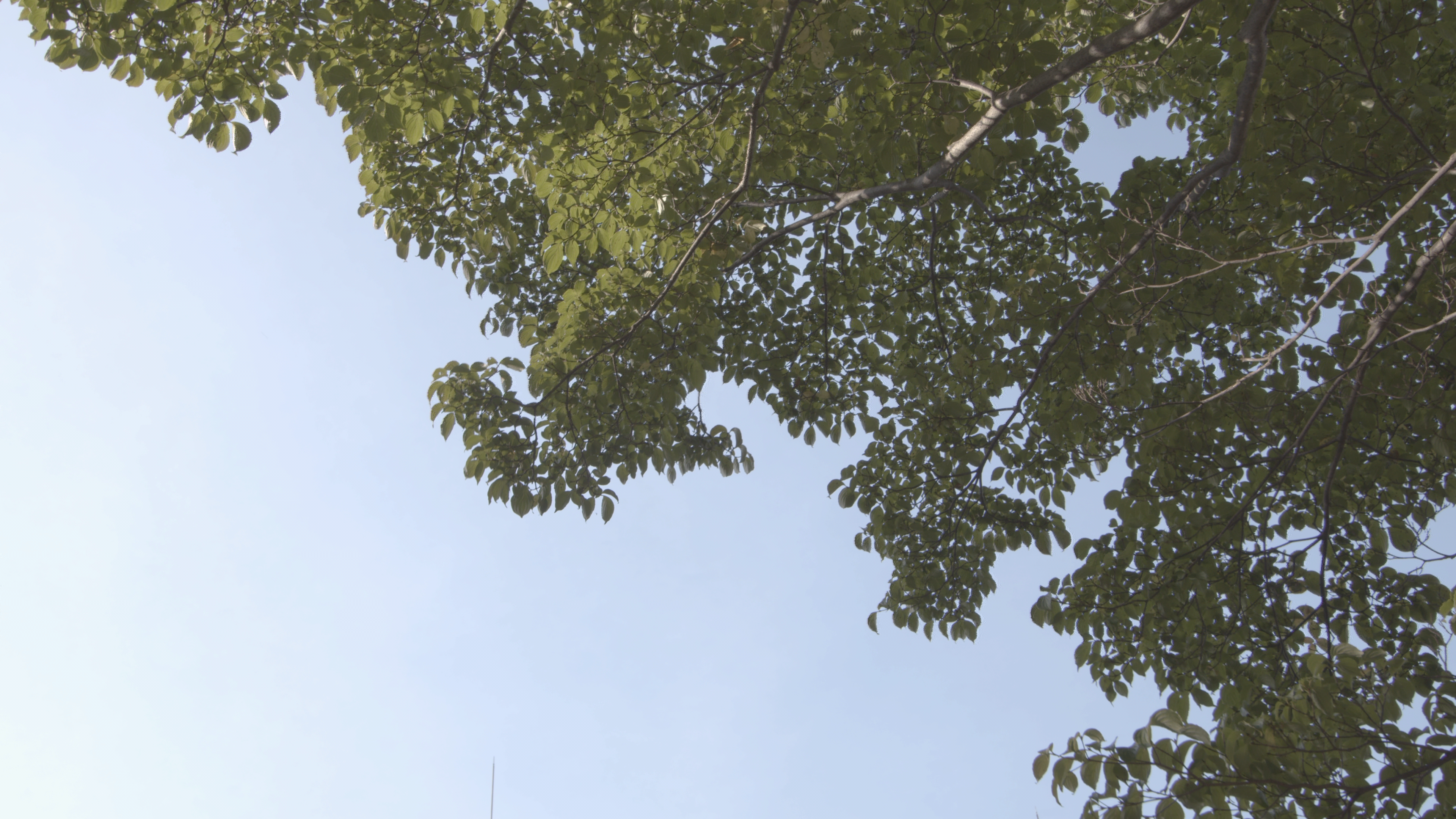

Supplement: Supplementary file 1 [file sensors-21-04602-s001.zip › supplimental_figures/Figure13/Figure7_encoded_second_eval_a.bmp]

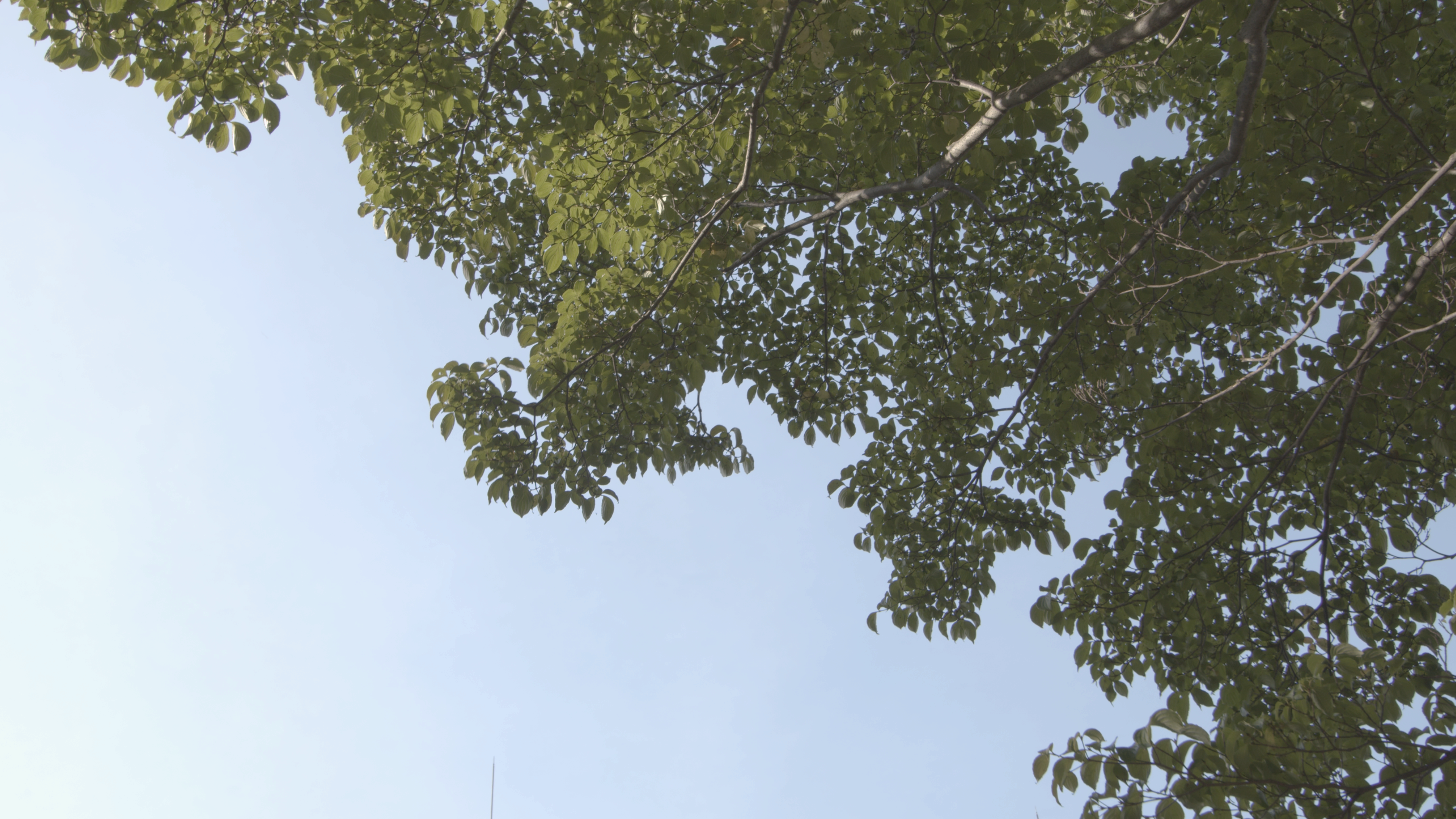

Supplement: Supplementary file 1 [file sensors-21-04602-s001.zip › supplimental_figures/Figure13/Figure7_original_second_eval_a.bmp]

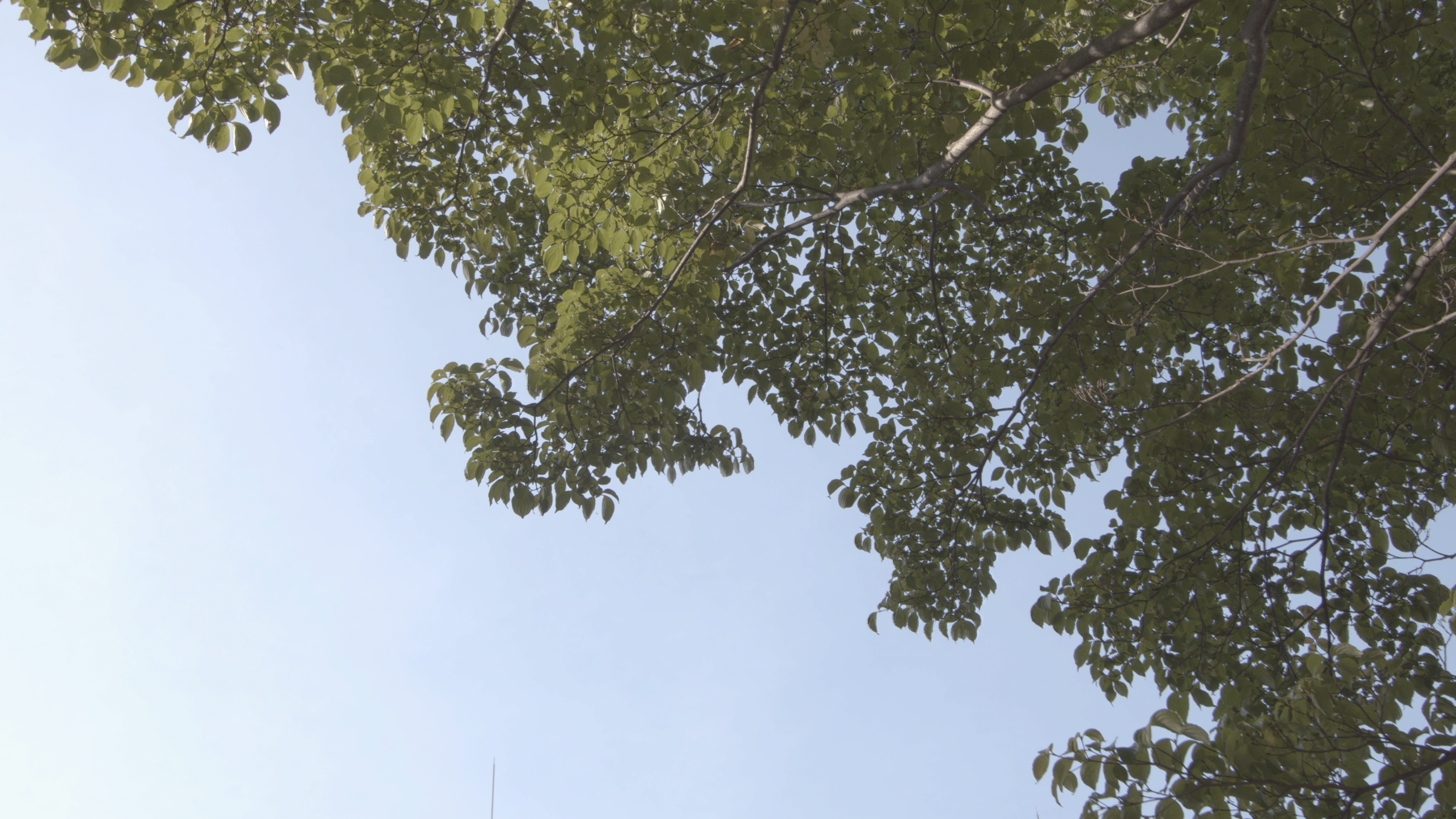

Supplement: Supplementary file 1 [file sensors-21-04602-s001.zip › supplimental_figures/Figure13/Figure7_encoded_first_eval_a.bmp]
